# Machine learning prediction of breast cancer local recurrence localization, and distant metastasis after local recurrences

**DOI:** 10.1038/s41598-025-89339-9

**Published:** 2025-02-10

**Authors:** Kristóf Attila Kovács, Csaba Kerepesi, Dalma Rapcsák, Lilla Madaras, Ákos Nagy, Anikó Takács, Magdolna Dank, Gyöngyvér Szentmártoni, Attila Marcell Szász, Janina Kulka, Anna Mária Tőkés

**Affiliations:** 1 Department of Pathology, Forensic and Insurance Medicine, Semmelweis University, Budapest, Hungary; 2 HUN-REN Institute for Computer Science and Control (SZTAKI), Budapest, Hungary; 3 National Institute of Oncology, Budapest, Hungary; 4 Department of Pathology and Experimental Cancer Research, Semmelweis University, Budapest, Hungary; 5 Department of Internal Medicine and Oncology, Semmelweis University, Budapest, Hungary

**Keywords:** Breast cancer, Local recurrences, Machine learning, Cancer, Computational biology and bioinformatics, Molecular biology, Oncology

## Abstract

Local recurrences (LR) can occur within residual breast tissue, chest wall, skin, or newly formed scar tissue. Artificial intelligence (AI) technologies can extract a wide range of tumor features from large datasets helping in oncological decision-making. Recently, machine learning (ML) models have been developed to predict breast cancer recurrence or distant metastasis (DM). However, there is still a lack of models that consider the localization of LR as a tumor feature. To address this gap, here, we analysed data from 154 patients including pathological, clinical, and follow-up data (with an average follow-up of 133.16 months) on both primary tumors (PT) and recurrences. By using ML methods we predicted the localization of LR and the occurrence of DM after LR. The performance (ROC AUC) of the best ML models was 0.75, and 0.69 for predicting LR in breast parenchyma, and surgical scar tissue, respectively, and 0.74 for predicting DM after LR. We identified recurrence localization, and the time elapsed between the detection of primary breast carcinoma and the recurrence, and adjuvant chemotherapy as the most important features associated with further DM. We conclude that combining traditional prognostic factors with ML may provide important tools in the risk assessment of patients with breast LR.

**Supplementary Information:**

The online version contains supplementary material available at 10.1038/s41598-025-89339-9.

## Introduction

Numerous studies focus on the importance of various tumor features considered to be associated with the occurrence of breast local recurrences (LR). Although multiple predictors of breast cancer recurrence risk and several algorithms have been analyzed and evaluated, to the best of our knowledge, no studies have employed ML models to predict the localization of breast cancer LR and subsequent distant metastases^1–3^.

Breast cancer recurrences can occur in the form of local or locoregional recurrences or manifest themselves as distant metastases. The different forms of the disease are associated with different outcomes and require different treatment approaches^4,5^. LR may occur within residual breast tissue or tissues of the chest wall or skin and may also affect the newly formed scar tissue. Based on the Maastricht Delphi Consensus, all recurrent invasive breast cancer or ductal carcinoma in situ (DCIS) in the ipsilateral breast or the skin and subcutaneous tissue on the ipsilateral thoracic wall are considered local events^6^.

According to important studies, LR are biologically inhomogeneous diseases; tumors with favorable and unfavorable prognosis could be separated^7^. LR could develop from *in loco* remaining cells of the primary tumors (PT) that survive adjuvant treatment (true recurrences) or may develop from a new clone as a second PT or may progress from an in situ carcinoma (the newly formed PT)^8–11^. Japanese researchers assessed four types of ipsilateral breast tumor recurrences after breast-conserving surgery. Those manifesting far from scar tissue or those that were close to the scar, originating from residual in situ carcinomas demonstrated better outcomes than true recurrences (from surviving local tumor cells)^12^. Comparing genetic features of true recurrences and newly formed primaries to the original PT or comparing different features of the recurrences occurring in different localizations could further clarify disease biology differences, but such studies are scarce^10^. Of the various molecular markers and tests designed to help predict the prognosis of breast cancer, the PIK3CA-AKT signaling pathway has been the focus of several studies. PIK3CA mutations are most common in HR-positive breast cancer, and recent data show that treatment-tolerant tumor cells with mutations in the PIK3CA-AKT pathway are more likely to have disease recurrence^11^.

Several studies have applied ML algorithms and integrated multiple tumor variables to improve diagnostic and prognostic accuracy. For example, Lou et al. by including several clinical characteristics, quality of care, and preoperative quality of life compared the performance of various ML algorithms to predict recurrence within ten years after breast cancer surgery^13^. Another recent study used three different ML models to forecast the recurrence of breast cancer across a very heterogeneous sample of patients^14^. Supplementary Table S1 presents a brief overview of recent publications using ML techniques and focusing on breast recurrences. While, ML models are more and more used in predicting breast cancer recurrence and distant metastases^2,14–20^, studies that consider the different localization of local recurrences (LRs) and distinct tumor features of both primary cancers and their corresponding recurrences are still lacking.

In our study, we compared several clinicopathological and molecular factors of primary breast carcinomas presenting with recurrences in the chest wall (CWR) (within scar tissue and chest wall skin) to those where recurrences were detected in breast parenchyma and investigated the outcome of these forms of disease relapses. By using four ML protocols we aimed to elucidate whether ML can predict the occurrence of LR at different localizations and with further distant metastases.

## Results

### Clinicopathological characteristics

The clinicopathological characteristics of patients are summarized in Table 1. All of the 154 patients in our study had some local recurrence (73, 63, and 18 cases in the remaining breast parenchyma, surgical scar tissue, and the skin of the chest wall, respectively). The patients were followed by 133.16 months on average (range 13 to 429 months). LR occurred on average 71.63 (range 3 to 278) months after initial diagnosis. In 46/154 (29.9%) cases LR was detected between 3 and 24 months (early recurrences), in 40/154 cases (26%) between 25 and 60 months (mid-term recurrences) and in 68/154 cases (44.2%) after a period of > 60 months (late recurrences). Recurrences were detected up to 23 years after primary diagnosis. Among the 154 patients, 33 became 10-year disease-free survivors as these late recurrences were detected after 10 to 23 years. During the relatively long follow-up time, distant metastases were observed in 91/154 (59.09%) cases and the average time from the diagnosis of LR to the first detection of distant metastases was 28.45 months (range 1–170 months).

**Table 1:** The distribution of the clinicopathological data of the patients. The impact of various factors on the occurrence of local recurrences in different localizations.

Features	All	Remaining breast parenchyma	Surgical scar tissue	Skin of the chest wall	Parenchyma vs. non-parenchyma
	Number of patients (%)				*p*-value
Total	154	73	63	18	
Age					ns.
< 35	7 (4.5)	1 (1.4)	5 (7.9)	1 (5.6)	(Cont.)
35–49	52 (33.8)	30 (41.1)	16 (25.4)	6 (33.3)	
50–69	79 (51.3)	39 (53.4)	31 (49.2)	11 (61.1)	
70≤	16 (10.4)	3 (4.1)	11 (17.5)	0	
LR time from PT (Rec time from PT (m))					0.001983
0–24 months	46 (29.9)	13 (17.8)	23 (36.5)	10 (55.6)	(Cont.)
25–60 months	40 (26.0)	20 (27.4)	18 (28.6)	2 (11.1)	
60 < months	68 (44.2)	40 (54.8)	22 (34.9)	6 (33.3)	
Distant metastasis (Met)					7.19E−07
Yes	91 (59.1)	28 (38.4)	47 (74.6)	16 (88.9)	(Yes vs. No)
No	63 (40.9)	45 (61.6)	16 (25.4)	2 (11.1)	
PT LVI (PT lymphovascular invasion)					0.01776
Yes	36 (50.0)	12 (34.3)	18 (62.1)	6 (75.0)	(Yes vs. No)
No	36 (50.0)	23 (65.7)	11 (37.9)	2 (25.0)	
n/a	82	38	34	10	
PT Histology (PT hist type)					ns.
NST	115 (79.3)	54 (80.6)	49 (81.7)	12 (66.7)	(NST vs. ILC)
ILC	17 (11.7)	5 (7.5)	7 (11.7)	5 (27.8)	
Other	13 (9.0)	8 (11.9)	4 (6.7)	1 (5.6)	
n/a	9	6	3	0	
PT HR positivity					ns.
HR pos	104 (75.3)	46 (74.2)	47 (79.7)	11 (64.7)	(HR pos vs. HR neg)
HR neg	34 (24.7)	16 (25.8)	12 (20.3)	6 (35.3)	
n/a	16	11	4	1	
PT Molecular subtype (PT subtype)					ns.
LumA	28 (25.4)	12 (25.0)	15 (31.2)	1 (7.1)	(LumB1 vs. TNBC)
LumB1	39 (35.5)	18 (37.5)	15 (31.2)	6 (42.9)	
LumB2	9 (8.1)	2 (4.2)	6 (12.5)	1 (7.1)	
HER2	6 (5.5)	3 (6.3)	3 (6.2)	0 (0.0)	
TNBC	28 (25.4)	13 (27.1)	9(18.8)	6 (42.9)	
n/a	44	25	14	4	
PT TNM T (PT TNM (T) sum y)					1.52E−06
PST	21 (16.4)	3 (5.1)	10 (18.9)	7 (46.7)	Cont.
Tx	2 (1.6)	0 (0.0)	1 (1.9)	1 (6.7)	
T1	47 (36.7)	34 (57.6)	11 (20.8)	2 (13.3)	
T2	42 (32.8)	21 (35.6)	17 (32.1)	4 (26.7)	
T3	11 (8.6)	1 (1.7)	9 (17.0)	1 (6.7)	
T4	5 (3.9)	0 (0.0)	5 (9.4)	0	
n/a	26	14	10	3	
PT TNM N (PT TNM (N) sum y)					3.37E−05
PST	21 (16.5)	3 (5.3)	11 (20.0)	7 (46.7)	Cont.
N0	56 (44.1)	37 (64.9)	15 (27.3)	4 (26.7)	
N1	31 (24.4)	12 (21.1)	17 (30.9)	2 (13.3)	
N2	16 (12.6)	5 (8.8)	10 (18.2)	1 (6.7)	
N3	3 (2.4)	0 (0.0)	2 (3.6)	1 (6.7)	
n/a	27	16	8	3	
Grade (PT grade)					ns.
Grade 1	31 (23.8)	14 (23.0)	13 (24.1)	4 (26.7)	(Cont.)
Grade 2	53 (40.8)	28 (45.9)	21 (38.9)	4 (26.7)	
Grade 3	46 (35.4)	19 (31.1)	20 (37.0)	7 (46.7)	
n/a	24	12	9	3	
PT Ki67 cat (PT Ki67%)					ns.
High (20+)	58 (55.2)	26 (53.1)	23 (52.3)	9 (75.0)	(high vs. low)
Low (0–19)	47 (44.8)	23 (46.9)	21 (47.7)	3 (25.0)	
n/a	49	24	19	6	
PT Surgery type (PT surgery type)					2.69E−14
BCS	89 (58.2)	65 (89.0)	17 (27.4)	7 (38.9)	(BCS vs. Mastectomy)
Mastectomy	64 (41.8)	8 (11.0)	45 (72.6)	11 (61.1)	
n/a	1	0	1	0	
PT Resection margin (PT resection margin (mm))					ns.
R0	100 (92.6)	53 (98.1)	37 (90.2)	10 (76.9)	(R0 vs. R1)
R1	8 (7.4)	1 (1.9)	4 (9.8)	3 (23.1)	
n/a	46	19	22	5	
PT Chemotherapy (PT chemotherapy)					ns.
Yes	84 (61.8)	36 (55.4)	35 (64.8)	13 (76.5)	(Yes vs. No)
No	52 (38.2)	29 (44.6)	19 (35.2)	4 (23.5)	
n/a	18	8	9	1	
PT Endocrine therapy (PT hormone therapy)					ns.
Yes	81 (60.0)	36 (56.3)	36 (66.7)	9 (52.9)	(Yes vs. No)
No	54 (40.0)	28 (43.8)	18 (33.3)	8 (47.1)	
n/a	19	9	9	1	
PT Target therapy (PT other therapy)					ns.
Yes	11 (8.0)	5 (7.7)	6 (10.9)	0 (0.0)	(Yes vs. No)
No	126 (92.0)	60 (92.3)	49 (89.1)	17 (100.0)	
n/a	17	8	8	1	
Radiotherapy (PT radiation therapy)					ns.
Yes	100 (75.2)	49 (79.0)	37 (68.5)	14 (82.4)	(Yes vs. No)
No	33 (24.8)	13 (21.0)	17 (31.5)	3 (17.6)	
n/a	21	11	9	1	
LR subtype switch (Rec subtype switch)					ns.
Yes	30 (36.1)	17 (38.6)	10 (30.3)	3 (50.0)	(Yes vs. No)
No	53 (63.9)	27 (61.4)	23 (69.7)	3 (50.0)	
n/a	71	29	30	12	
Multiple LR					ns.
Yes	51 (33.1)	19 (26)	28 (44.4)	4 (22.2)	(Yes vs. No)
No	103 (66.9)	54 (74)	35 (55.6)	14 (77.8)	

*We used *t*-tests for the continuous variables (‘Cont.‘) and Fisher exact test for the binary comparisons. The number of missing values (“n/a”) is also indicated.

*BCS* breast conserving surgery, *HR* hormone receptor, *LR* local recurrence, *PST* primary systemic therapy, *PT* primary tumor.

We also collected the features of the PT for the 154 LR cases (Table 1). The median age of patients at the PT diagnosis was 54 years (range, 30–91 years). Most patients (70%) had stage I or stage II PT. Of the 138 cases with known hormone-receptor (HR) status 104 patients (75.3%) had HR-positive and 34 (24.7%) had HR-negative disease. The detailed subtype distribution is presented in Table 1. Of the fifteen of the well-documented HER2-positive cases, eleven patients received HER2 targeted therapy for primary breast cancer (HER2-directed therapy was not available until 2009 in adjuvant or neoadjuvant settings). All patients underwent curative surgery: 89 (58.2%) underwent breast-conserving surgery, 64 (41.8%) had total mastectomy and in one case no data was available. Of the 89 patients treated with breast-conserving surgery known data about the applied radiotherapy was available in 73 cases: 60/73 (82.1%) underwent adjuvant radiotherapy. In the group of patients who underwent mastectomy and had available data, 67.2% (39/58) received adjuvant radiotherapy. Postoperative pathology revealed IDC-NST in 79.3% of the cases, ILC in 11.7% and other histotype in 9.0%. Ipsilateral breast parenchyma recurrences were documented in 73/154 (47.4%) cases, while CWR in 81/154 (52.5%) cases. Of the CWR cases 63/81 (77.7%) occurred within the breast surgical scar and 18/81 (22.2%) as skin recurrences in the breast or in the thoracic wall.

### Machine learning prediction of breast cancer local recurrence localization

We used ML methods to predict the localization of LRs based only on the PT features. We randomly selected 124 cases for training and 30 cases for testing the methods. We tried several classification protocols (XGBoost with and without feature selection, as well as random forest) by cross-validation on the training set (see the “Methods” and Supplementary Table S2). After the optimization process on the training set, we tested the best models on the independent testing set. We examined different prediction tasks independently and reported the ones having remarkable performance (~ 0.7 ROC AUC on the testing set).

**Fig. 1:**
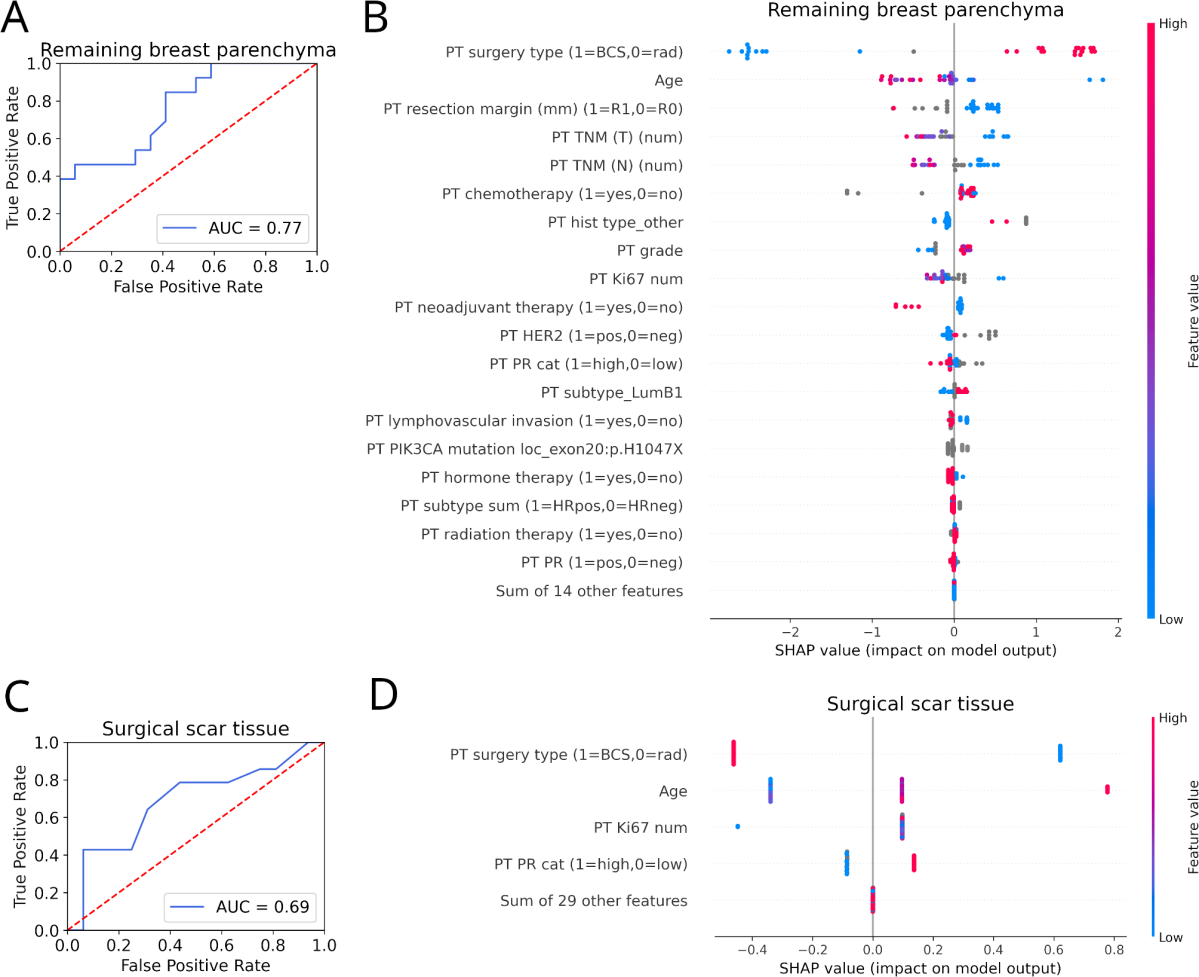
Machine learning prediction of breast cancer local recurrence localization. (**A**) Prediction performance (ROC AUC) of the remaining breast parenchyma localization recurrence versus any other examined location (i.e., surgical scar tissue or skin of the chest wall) using primary tumor (PT) features. (**B**) The most important features and their impact on the output for the best model predicting the remaining breast parenchyma localization of the LR. Cases on the right or left side of the diagram indicate a positive or negative impact of the given feature on the prediction, respectively (each dot represents a test case). The further a point is from zero, the more important the feature is. The features are sorted by importance (i.e., impact in deciding the localization of recurrence). Blue dots indicate low, and red dots indicate high values of the given variable for the given test case (grey dots indicate missing values). In the case of binary variables, we indicated which case is marked with a 0 or 1 value. (**C**) Prediction performance of surgical scar tissue localization vs. any other examined location (i.e., the remaining breast parenchyma or the skin of the chest wall). (**D**) The most important features and their impact on the model output for the best model predicting the surgical scar tissue localization of LR. *BCS* breast conserving surgery, “*cat*” category, “*hist*” histology, *LR* local recurrence, “*num*” number, *PST* primary systemic therapy, *PT* primary tumor, “*rad*” radical surgery, *TNM* Tumor/Node/Metastasis.

The performance of predicting the remaining breast parenchyma as an LR localization versus the remaining localizations (i.e., surgical scar tissue, and the skin of the chest wall) was 0.77 (Fig. 1A). The model used 19 features, and the most important features of the prediction were the surgery type (breast-conserving or radical), age at the diagnosis, and the resection margin (Fig. 1B). The performance of predicting the surgical scar tissue as a LR localization versus the remaining localizations (i.e., remaining breast parenchyma, and the skin of the chest wall) was 0.69 (Fig. 1C). The model used only four features, and the most important features of the prediction were again the surgery type, and age at the diagnosis, however, Ki67 positivity ratio and progesterone receptor (PR) status were also important (Fig. 1D).

### Machine learning prediction of distant metastasis after local recurrences

**Fig. 2:**
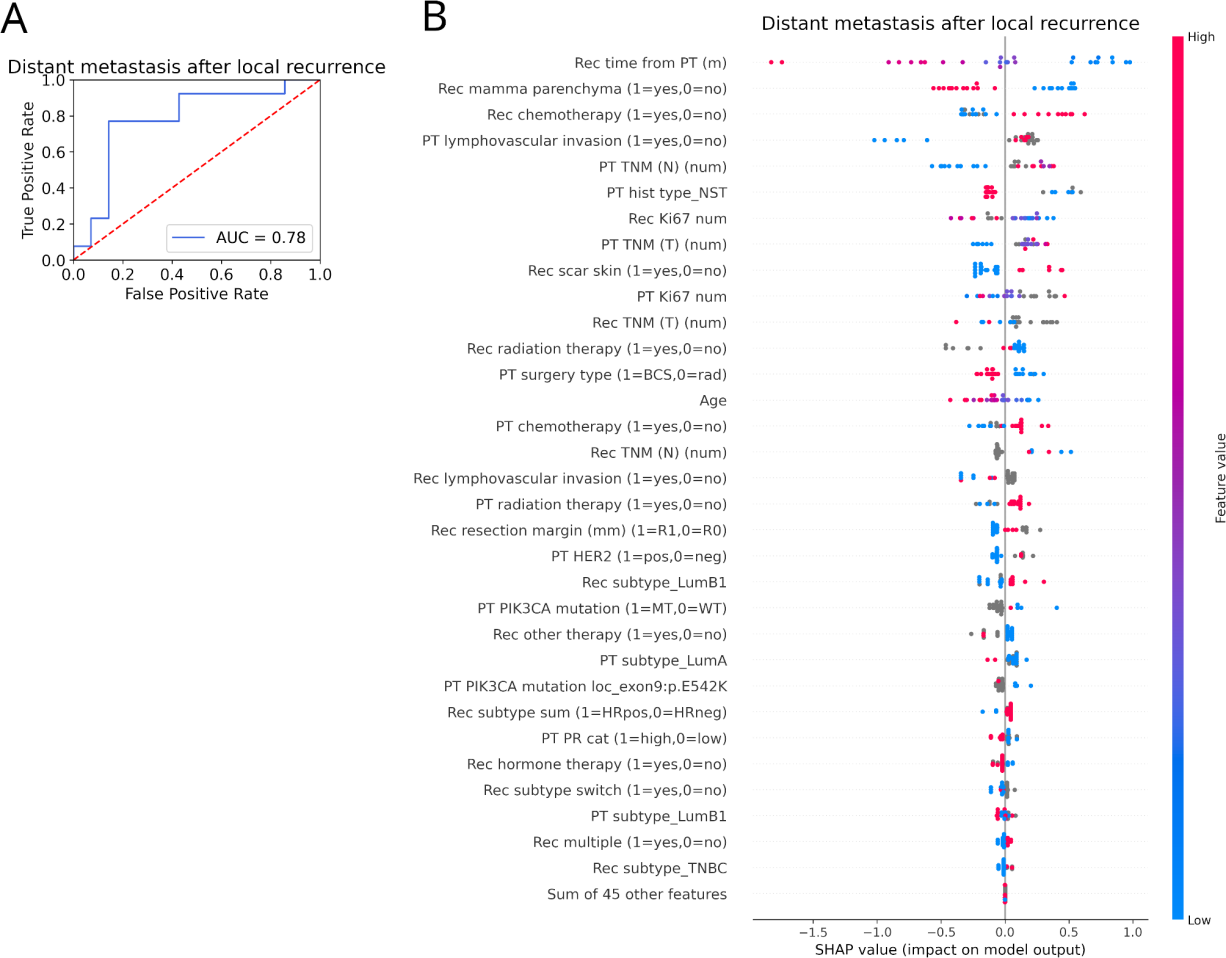
Machine learning prediction of distant metastasis after local recurrences. (**A**) Prediction performance (ROC AUC) of the DM after LR. (**B**) Most important features their impact on the model output for the best model predicting the occurence of DM after LR. Cases on the right or left side of the diagram indicate a positive or negative impact of the given feature on the prediction, respectively (each dot represents a test case). The further a point is from zero, the more important the feature is. The features are sorted by importance of the prediction. Blue dots indicate low, and red dots indicate high values of the given variable for the given test case (grey dots indicate missing values). In the case of binary variables, we indicated which case is marked with a 0 or 1 value. *BCS* breast conserving surgery, “*cat*” category, “*hist*” histology, *LR* local recurrence, “*m*” months, “*neg*” negative, “*num*” number, *PT* primary tumor, “*rad*” radical surgery, *NST* no special type, *pos* positive, *TNBC* triple negative breast carcinoma, “*Rec*” recurrence, *TNM* Tumor/Node/Metastasis.

We also predicted the occurrence of DM after LR based on PT and LR features. We used the same train-test split and machine learning methods as above. The performance of predicting DM after LR was 0.78 (Fig. 2A). The model used 32 features, and the most important features of the prediction were the elapsed time between the detection of PT and the occurrence of LR, the localization of the LR (in the remaining breast parenchyma or not), and the therapy of the LR (chemoterapy or not). While the higher elapsed time between PT and LR, as well as, the localization of the LR in the remaining breast parenchyma decreased the chance of DM after LR, the chemoterapy of the LR increased the chance of it (Fig. 2B).

### Survival analysis of distant metastases after local recurrences

**Fig. 3:**
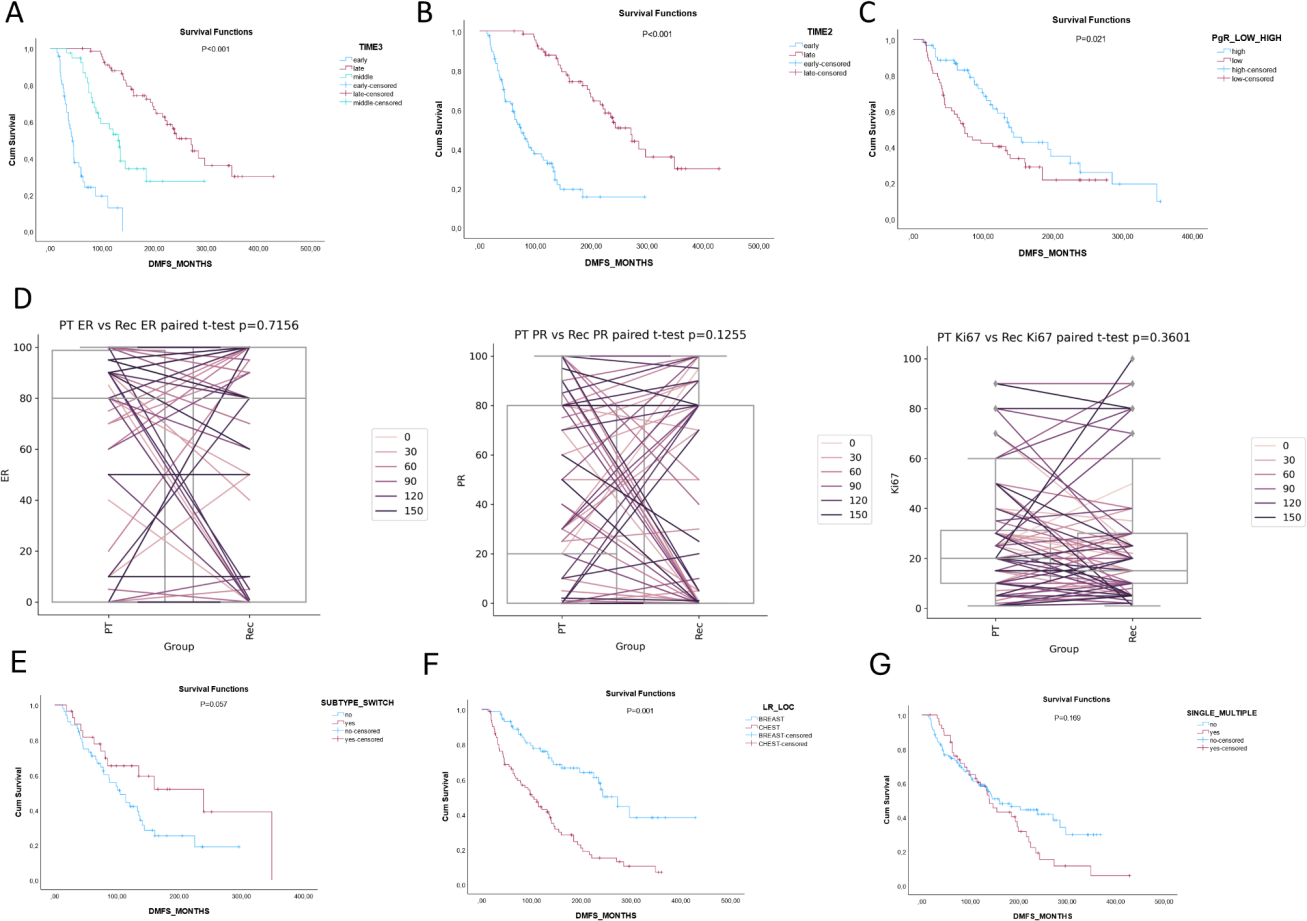
Survival analysis of distant metastases after local recurrences. (**A**,**B**) Distant Metastasis-Free Survival (DMFS) separated by the time of recurrences occurring in ≤ 24 months after the diagnosis of primary breast carcinoma. (**C**) DMFS separated by low or high PR expression in primary breast carcinomas (<20%). (**D**) Changes in ER, PR (PR), and Ki67 protein expression in primary breast carcinomas (PT) vs. corresponding recurrences (Rec). (**E**) DMFS for a switch or not switch in the subtype. (**F**) DMFS separated by different localizations of the recurrences. (**G**) DMFS separated by the occurrence of single or multiple local recurrences.

Kaplan–Meier analyses also showed significant differences in the occurence of distant metastasis based on several factors like the time elapsed between the detection of primary breast carcinoma to the occurrence of recurrence. Early recurrences are significantly associated with worse DMFS (*p* < 0.001) (Fig. 3A, B). Low PR expression in primary breast carcinomas (< 20%) was also associated with shorter DMFS (*p* = 0.021) (Fig. 3C). Patients presenting with local recurrence in the chest wall faced a higher incidence of distant metastases compared to the patients presenting with LR in breast parenchyma (*p* = 0.001) (Fig. 3F). However, no association was detected between switch in subtype (between primary breast carcinomas and corresponding LR) and occurrence of distant metastases (*p* = 0.057) (Fig. 3E). Subtype definition in both primary and recurrent pairs was available in 83 cases. Of these subtypes concordance between the PT and paired local recurrence occurred in 53/83 cases. Estrogen receptor (ER), progesterone receptor (PR), and Ki67 concordance between primary breast carcinoma and corresponding local recurrent cases was analyzed case by case. By comparing the expression of the above-mentioned three markers in primary vs. corresponding LR no statistically significant changes were detected (Fig. 3D).

Multiple LRs were diagnosed in 51/154 (33.11%) cases. Nevertheless, the occurrence of second or multiple local recurrences was not significantly associated with shorter DMFS (*p* = 0.169) (Fig. 3G). The ratio of second or multiple recurrences was higher in the group of patients diagnosed with surgical scar recurrences (28/63 cases (44.44%)) compared with the group of patients where the first recurrences were detected in breast parenchyma (19/73 cases (26.02%)). Of the clinicopathological features of primary breast carcinomas presenting later with two or multiple local recurrences the high ratio of LUMB1 subtype (22/35 (62%)) is remarkable compared to the group of patients presenting with a single recurrent tumor, where only 26/85 of the cases (30.58%) presented LUMB1 subtype.

### PIK3CA mutations

PIK3CA mutation analysis was performed in 34 pairs (primary breast carcinoma and corresponding LR). Pathogenic mutations were detected in 14/34 (41.1%) of the analyzed PTs and in 12/34 (35.2%) in the corresponding recurrent lesions. The most frequent PIK3CA mutations in primary breast carcinomas were detected in exon 9 (E542K/E545K) (10/14 cases) followed by mutations in exon 20 (3/14 cases) (H1047XR/H1047L) and by mutation in exon 7 (1/14 cases). In LR the frequency of mutations was the following: mutations in exon 9 in 8/12 cases, mutations in exon 20 in 3/12 cases, and mutations in exon 7 in 1/12 cases. There were instances where recurrences were wild type in patients with PIK3CA mutant PTs and vice versa. In the combined analysis, 3 patients changed genotype from wild type to mutant compared to 4 patients who lost mutations in the recurrences (Table 2). Of the 14 primary breast tumors presenting with PIK3CA mutations distant metastases were diagnosed in 10/14 cases whereas in wild type tumors, this ratio was 11/20.

**Table 2:** Cases presenting with discordant PIK3CA mutation in primary breast carcinomas compared to local recurrent pair.

Case	Surgery	Tumor	Location	Histology	IHC type	*P*-*R* interval (months)	PIK3CA status	Mutation type
1	Mastect.	Primary	Right MUQ + MLQ (double tumor)	Mixed NST + ILC	Lum A	48	MT	exon9, p.E545X
		LR	Scar	NST	LumA		MT	exon20, p.H1047X
2	Mastect.	Primary	Right central	NST	LumA	111	WT	
		LR	Multiple scar LRs	NST	LumA		MT	exon9, p.Q546X
3	Mastect.	Primary	Left diffuse	ILC	LumA	32	MT	exon9, p.E542K
		LR	Multiple scar LRs	ILC	LumA		WT	
4	Mastect.	Primary	Left diffuse	NST	LumB1	21	MT	exon9, p.E542K
		LR	Scar	NST	LumB1		WT	
5	BCS	Primary	Left central	NST	LumA	41	MT	exon20, p.H1047X
		LR	Left central	NST	LumA		WT	
6	BCS	Primary	Left MUQ	NST	LumA	46	MT	exon9, p.E542K
		LR	Left MQ	NST	LumA		WT	
7	BCS	Primary	Right LUQ	NST	LumA	56	WT	
		LR	Right LQ	NST	LumA		MT	exon9, p.E545X
8	BCS	Primary	Left LUQ	Micropapillary	LumA	94	WT	
		LR	Axillary scar	Micropapillary	LumA		MT	exon20, p.H1047X

*Mastect* mastectomy, *BCS* breast-conserving surgery, *LR* local recurrence, *MQ* medial quadrant, *LQ* lateral quadrant, *MUQ* medial upper quadrant, *MLQ* medial lower quadrant, *LUQ* lateral upper quadrant.

## Discussion

In this study, we aimed to identify important key breast tumor characteristics for predicting breast LR localization and the occurrence of distant metastases by considering the available patient-level data and ML algorithms. This is especially important since LR of the disease is seen in 5–20% of breast cancer patients, even after adequate primary treatment^21–25^ and recurrences can manifest across various locations, spanning extensive periods^23^.

Several studies experienced elevated risk of distant metastases and death in patients with LR. According to our results, using ML algorithms we found that „anatomical” (location of the recurrent tumor) and „temporal” (time from primary diagnosis to local recurrence) features are the most important characteristics of LR that predict organ metastases. We also experienced a positive correlation between induction of adjuvant chemotherapy and distant metastases following LR implying that LR can be and indicator of preserved metastatic potential despite the applied chemotherapy so a different, targeted type of systemic therapy could be more beneficial.

After breast-conserving surgery, the most common localization of relapses is within the remaining breast parenchyma, while chest wall recurrences occur mostly after post-mastectomy^24,25^. Chest wall skin recurrences are seen mainly within surgical scar tissue, or within its surroundings, but also may manifest as non-scar related skin lesions^26^. There are several theories about why scar recurrences are a frequent type of local relapses. Remaining tumor cells after surgery or cells from surgical spill might be the origin of tumor regrowth or tumor stem cell activation, both enhanced by microenvironment changes through wound healing. Several studies proved that surgical tissue injury, followed by reparation provides an optimal milieu for tumor growth^27–29^.

Differences in local relapse localizations are most probably due to their different biologies, thus their prognostic significance differs and management strategies should evolve accordingly^30^. It is always a challenge to determine the most significant tumor features associated with tumor progression.

ML has increasingly been applied in pathology and oncology and several prediction models were developed to increase diagnostic and prognostic accuracy, and to predict tumor progression^16,28,31,32^ (Supplementary Table S1). Here, we used the features of primary breast carcinomas and of LR in the task of predicting DM after LR while in the remaining prediction tasks when the target variable was a recurrence feature, we used only the primary tumor features. Different best-performing machine learning methods were employed for classification and the model was selected based on the ROC curve. The SHAP (SHapley Additive exPlanations)^2,16^ method was used to examine the importance of the input features of the machine learning models. The SHAP method also provides us the number of features that have zero (or near-zero) impact on the predictions. In our analysis, this number was 14, 29, and 45 for the prediction of remaining breast parenchyma localization, surgical scar tissue, and DM after LR, respectively. This means that excluding these features would not considerably change the prediction performance. In contrast including more features, more samples, filling in more missing values may improve prediction performance. It would be worst to try it in future studies.

Studies using machine learning algorithms to predict breast cancer recurrence found that XGBoost and SHAP are suitable tools in the investigation of breast cancer recurrence. Gonzalez-Castro et al. have found that of the five evaluated algorithms to predict 5-year cancer recurrence the XGBoost model yielded the best performance^33^. A very recent study used eleven different machine learning algorithms to select the best model for predicting breast cancer recurrence^16^. To rank the feature importance they used SHAP values and they found that AdaBoost algorithm had the best prediction performance for successfully predicting breast cancer recurrence.

In our CWR cohort, 63 scar relapses and 18 non-scar-related skin relapses occurred. In scar relapses, we could not examine the exact location of the tumor within the scar (i.e. which layer of the skin was affected). Zhou et al. concluded that skin involvement in CWRs is an unfavorable prognostic sign supporting the theory that skin involvement in a surgical scar relapse could rather underline the dissemination capacity of a tumor, and is not merely a tumor regrowth from residual tumor cells^34^.

Of the several tumor features analysed in different studies predicting local recurrences or any second breast cancer, patients age at diagnosis, tumor size, and tumor grade are the most common features identified also in our study^32,35^.

As in many studies we used predictors and tumor features readily available from medical records. Notable difference between our study and those in the literature focusing on LRs is that our study focuses on the importance of recurrent tumor localization also considering the impact of LRs on further tumor progression as well as the impact of LR on the occurrence of multiple local recurrences. Special attention was also given to the differences in biomarkers between the PT and LR in predicting tumor dissemination.

Accordingly, we have found that early relapses (LR less than 2 years after initial diagnosis) were more commonly associated with systemic recurrences (i.e. DM). Early relapses and disseminated disease are associated with worse disease biology: these cases are usually TNBCs. Late relapses—those manifesting five years after initial diagnosis—are mainly ER positive tumors, with low grade and low stage. Pan et al. have found that even after 5 years of adjuvant endocrine therapy, early-stage ER-positive breast cancer still had a persistent risk of recurrence and death from breast cancer for at least 20 years after the original diagnosis^36^. In our series 56% of the cases presenting with early recurrences (<24 months) were HR+ raising the necessity of differentiated therapy in different HR+ breast carcinomas. A recent study showed that among women with PR-positive primary breast cancer compared to those with PR-negative primary cancer the risk of death following local recurrence was lower (ten-year mortality 30% vs. 60%)^37^.

Few data about very late relapses are available. In our study, 20.77% of patients had LR 10 years or more after initial diagnosis. The majority of these (83.33%) were HR + tumors. According to the Danish Breast Cancer Group clinical database, 12.77% of LR occur more than 10 years after initial diagnosis^23^.

It is partly unknown whether differences in biomarkers between the PT and LR have a role in predicting tumor dissemination. According to our results differences in tumor biology were not significantly related to systemic relapses nor showed association with the location of LR. Okumura et al. could not demonstrate a significant association between changes in the expression of ER and HER2 and survival, but a change of Ki67 expression predicted distant metastases^38^. Tumor genotype analysis could establish similarities between PTs and LR. The most assessed genetic alteration especially in ER positive BCs is PIK3CA mutation. The prevalence of PIK3CA mutation varies according to the subtype and stage of breast cancer and most studies compare PT PIK3CA mutational status to that of distant metastases^39,40^. Jensen et al. examined PIK3CA mutational status differences of PTs and their paired metastases and investigated whether these differences could predict survival. According to their study those patients whose metastases demonstrated PIK3CA mutation, initially not present in their PT did not have more aggressive disease, but later recurrences^41^. There is only scarce data available about PIK3CA mutational differences between PTs and LR. Nakagomi et al. analysed primary breast cancers and paired LR cases. Residual recurrence and double primary categories were created according to the comparison of their histopathological features and targeted deep sequencing analysis focusing on breast cancer driver genes. Regarding PIK3CA mutation status a much higher proportion of residual recurrences showed mutated genotypes compared to double primaries (85% vs. 25%), so cases with mutations in the PIK3CA-AKT pathway were more likely to have recurrent disease^11^. We found that 41.1% of PTs showed PIK3CA mutations as opposed to 35.2% in LR. Among the 14 mutant recurrent cases, 6 occurred within the breast parenchyma and 8 were CWRs.

Another important feature of LR is that it is usually not a singular event. According to a study of Lim et al. in 20.7% of patients with LR, another relapse occurred^42^. In our cohort of patients, 33.11% demonstrated multiple LR. Geurts et al. demonstrated that 80% of patients with LR had distant metastases if the second event followed the first LR within a year. In our study, 72.5% of patients with multiple events of LR had distant metastases^43^.

We acknowledge that our study has certain limitations. The majority of the cases are present with a long follow-up period, and we cannot account for eventual differences in therapeutic protocols during this long time interval. The cases needed to be further categorized based on subtype and important clinicopathological characteristics resulting in a smaller number of cases for some statistical analyses. As a characteristic of real-world data, some tumor features belonged to an unknown category. It is important to note that while validating our model using an external dataset would be beneficial, each study comes from different sources with variable characteristics. We have not yet identified studies that accurately assess the localization of recurrences in conjunction with various tumor features. Additionally, the significance of multiple local recurrences is rarely addressed in the existing literature.

## Conclusions

Local recurrences (LR) in breast cancer may demonstrate heterogeneous clinical and pathological features. No prediction model exists so far for breast cancer LR localizations and only a few studies predicted the occurrence of distant metastases based on several features of primary breast tumors and of corresponding LR. Our results demonstrate that the localization of LR matters and has a prognostic significance. Skin involvement—not only of the chest wall skin but also that of the surgical scar—demonstrates increased dissemination capacity of the tumor and it is also associated with an unfavorable outcome. Traditional prognostic factors and ML tools, combined with tumor genetic analyses could establish predictive models that based on high patient number cohort analysis may provide data in the risk assessment of patients with LR.

Our method, utilizing ML models, offers an approach to risk assessment, enabling the selection of patients who may benefit from carefully planned systemic treatments at the time of LR. Given that LR might predict the high probability of cancer dissemination, early identification of these patients may lead to improved treatment outcomes.

## Methods

Our initial cohort consisted of 448 breast carcinoma cases diagnosed with local recurrences. As the major aim of the study was to analyze the prognostic significance of the localization of ipsilateral recurrences and several other aspects of LR we focused on cases where as many as possible clinicopathological parameters were available for primary breast carcinomas as well as for the corresponding LR. Accordingly, cases with several missing pathological and/or clinical data of the PT and/or of relapses were excluded from the initial cohort. Finally, our retrospective study consisted of 154 primary breast carcinomas and corresponding LR pairs diagnosed between 1984 and 2018. The study was conducted in accordance with the Declaration of Helsinki. Clinicopathological data of the patients were obtained from the files of the Department of Pathology, Forensic and Insurance Medicine, Semmelweis University Budapest and from the Semmelweis University Health Care Database with the approvals of the Hungarian Medical Research Council (ETT-TUKEB 14383/2017 and 17781-3/2024). It is to be mentioned that starting from the year 2000 in the case of equivocal HER2 immunohistochemical result (score 2+) HER2 FISH was performed whereas immunohistochemistry data for ER, PR detection have been available since 1992. Of the 154 cases 8/154 PTs were detected before 1992, and 38/154 before 2000.

LR was defined according to the Maastricht Delphi Consensus of recurrence in breast cancer research^6^. Local recurrence-free survival was defined as the time from primary breast cancer diagnosis to the occurrence of the first LR diagnosis. Distant metastasis-free survival (DMFS) was defined as the time from the date of primary breast cancer diagnosis to the occurrence of the first DM. All patients were followed up until the date of death or until November 30, 2022.

The most important clinicopathological data are recorded in Table 1. Among them are the patient’s age at diagnosis, histological grade, pathologic tumor size (pT), nodal involvement (pN), LVI, resection margins, applied oncological treatment regimens and surrogate breast carcinoma subtype as defined based on four immunohistochemical markers (estrogen receptor (ER), progesterone receptor (PR), Ki67 index and HER2) according to the 2013 St. Gallen Consensus Conference recommendations^44^. Luminal A (LUMA) tumors are defined as ER and PR positive, HER2 negative, Ki-67 “low” (Ki-67 < 20%) tumors, Luminal B-HER2 negative (LUMB1) tumors as ER positive, HER2 negative and Ki-67 “high” (≥ 20%) and/or PR “negative or low” (PR cut-point = 20%), Luminal B-HER2 positive (LUMB2) as ER positive and HER2 overexpressed or amplified and HER2 positive/hormone receptor (HR) negative, and, triple negative breast carcinomas (TNBC) as HR and HER2 negative.

### PIK3CA mutation analyses

PIK3CA mutation status was determined in 34 pairs of PTs and corresponding LR with the Cobas^®^ PIK3CA Mutation Test (Roche, Basel, Switzerland), following the manufacturer’s instructions. The test can detect mutations in exon 1 (p.R88Q), exon 4 (p.N345K), exon 7 (p.C420R), exon 9 (p.E542K, p.E545A/G/K, p.E545D (only the nucleotide change c.1635G> T), p.Q546E/K/L/R) and exon 20 (p.M1043I (only the nucleotide change c.3129G> T), p.H1047R/L/Y, p.G1049R) of the PIK3CA gene when the mutant allele frequency is 5% or greater. 50 nanograms of template DNA was used in 25 µl volume in each PCR reaction. Amplification was carried out in the real-time PCR-based Cobas Z 480 analyzer (Roche, Basel, Switzerland). All samples were run in triplicates. Results were interpreted using Cobas^®^ 4800 System Software version 2.0 (Roche, Basel, Switzerland).

### Data preparation for machine learning and statistics

We performed one-hot encoding for the categorical variables that had three or more distinct values. After this process, all of the categorical variables became binary with 0 (i.e., false) or 1 (i.e., true) values. This is a regular pre-processing method preceding training by machine learning algorithms in the case we have categorical variables with more than two distinct categories. The final data table contained 154 rows (patients) and 84 columns (training features and target variables).

### Machine learning

Before training, we randomly split the feature table into ~ 80% training (*n* = 124) and ~ 20% test set (*n* = 30) to have enough patients for the training and the testing process. We experimented with several classification protocols (XGBoost^33^ with and without feature selection, as well as random forest) and optimized the hyperparameters by 5-fold cross-validation (repeating 10 times) on the training set (Supplementary Table S2). After the optimization process on the training set, we tested the best models on the independent testing set. We examined the different prediction tasks independently. In the prediction task of DM after LR, we considered only the cases when the metastasis was not the first recurrence. We used PT and LR features in the task of predicting DM after LR while in the remaining prediction tasks when the target variable was a recurrence feature, we used only the PT features. For each case, we performed a parameter optimization on the training set by 5-fold-cross-validation repeated 10 times. In the grid search, we considered 24 cases total with the parameter setting maximum depth = 1, 2, 3, 5, and the number of estimators (i.e., decision trees) = 1, 2, 5, 10, 20, 100. We used this process through four machine learning protocols: (i) using XGBoost classification and selecting the best model (the model with the highest mean ROC AUC) of the grid search, (ii) using XGBoost classification and selecting the best simple model (with maximum depth = 1) of the grid search, (iii) selection of the features by the best model of the XGBoost then re-train XGBoost only with the selected features, (iv) using random forest classification and selecting the best model of the gridsearch. Finally, we retrained a final model with the selected parameters on the entire training set. We examined the final models having remarkable performance (at least ~ 0.7 ROC AUC on the testing set). This criterion was satisfied in the prediction task of the remaining breast parenchyma (best XGBoost model; max depth = 3, number of estimators = 20), surgical scar tissue (best simple XGBoost model; max depth = 1, number of estimators = 10), and DM after LR (best XGBoost model; max depth = 3, number of estimators = 20) (Supplementary Table S2).

### Model explanation

The SHAP (SHapley Additive exPlanations) method^2,16^ was used to examine the importance of the input features of the machine learning models. SHAP values show how each feature affects the final predictions (impact on the model output) and the importance of each feature compared to others. The highest the sum of the absolute impacts of a feature the more important is the feature in the prediction^45^.

### Missing values

While some clinicopathological data (localization of the LR, LR time from PT, multiple LR, DM, and age) are available for all the 154 examined patients, others may contain missing values (i.e., n/a). In most of the cases, the proportions of the missing values are lower than 20% (Table 1). We did not use any missing value imputation techniques as the machine learning model implementations that we used can handle missing values as special values. RF and XGBoost build the missing values into the inferred models as rules of the decision trees.

### Statistical analysis

When we examined the relationship of two variables, we performed a Fishers’s exact test when both variables were binary, or a two-sided *t*-test when one variable was binary and the other one was continuous, or we calculated a Pearson’s correlation coefficient (‘r’) when both variables were continuous. P-values below 0.05 were interpreted as significant. For the statistical analysis, we used the fisher_exact, ttest_ind, pearsonr functions from the stats module of SciPy (v1.7.1). For all data analysis, we used Python (v3.9.7). Distant metastasis free survival (DMFS) was evaluated using Kaplan–Meier survival curves and the log-rank test was used to compare DMFS between the two groups.

## Electronic supplementary material

Below is the link to the electronic supplementary material.

Supplementary Material 1

Supplementary Material 2

## Data Availability

The authors declare that the data supporting the findings of the presented study are available within the article. Detailed clinical data of individual patients cannot be provided due to ethical restrictions but are available upon reasonable request from the corresponding author. The most important clinicopathological data are presented in Table 1.
