# Supplementary material for: Machine learning prediction of breast cancer local recurrence localization, and distant metastasis after local recurrences
Source: Sci Rep. 2025 Feb 10;15:4868. doi: 10.1038/s41598-025-89339-9 (PMC11811162; doi:10.1038/s41598-025-89339-9)
Supplement: Supplementary file 1 — Supplementary Material 1 [file 41598_2025_89339_MOESM1_ESM.doc]

**Supplementary Table S1. Key studies focusing on breast cancer recurrence risk prediction by using**

**machine learning models.**

|  | **Study size** | Title | **ML algoritms** | **Main results** |
| --- | --- | --- | --- | --- |
| 1 | literature review^15^ | Evolution of breast cancer recurrence risk prediction: a systematic review of statistical and machine learning–based models | Traditional models, ensemble models, and deep learning models | Deep learning and ensemble learning provide the most accurate predictions |
| 2 | 198151 nonrecurring and 47 recurring cases^46^ | Prediction time of breast cancer tumor recurrence using machine learning | SVM, DT, and RF | The prediction models are capable of predicting the time as accurately as 1 year for the tumor to reappear in malignant patients. |
| 3 | 1629  212 recurrence cases and 1417 non-recurrence cases^2^ | Clinical decision support tool for breast cancer recurrence prediction using SHAP value in cooperative game theory | Bagging, RF, Extra [Trees](https://www.sciencedirect.com/topics/biochemistry-genetics-and-molecular-biology/tree), AdaBoost, Gradient Boosting, ensemble learning | By utilizing four key factors, tumor size, clinical stage III, number of [lymph node metastases](https://www.sciencedirect.com/topics/medicine-and-dentistry/lymph-node-metastasis), and age, the decision support tool for predicting breast cancer recurrence achieved significant improvements |
| 4 | 529 primary breast carcinomas^17^ | A machine learning ensemble approach for 5- and 10-year breast cancer invasive disease event classification | Novel ensemble machine learning classification approach (voting among multiple models) | Improvement compared to the performances obtained by the baseline original model |
| 5 | 217 standard breast cancer recurrence prediction cases (84 positive cases and 133 negative cases)^20^ | A case-based ensemble learning system for explainable breast cancer recurrence prediction | XGBoost, [LR](https://www.sciencedirect.com/topics/computer-science/logistic-regression), KNN (K = 5), SVM, C4.5 [DT](https://www.sciencedirect.com/topics/biochemistry-genetics-and-molecular-biology/decision-trees), [RF](https://www.sciencedirect.com/topics/computer-science/random-decision-forest), MLP, and [gradient boosting](https://www.sciencedirect.com/topics/computer-science/gradient-boosting) [decision tree](https://www.sciencedirect.com/topics/computer-science/decision-trees) (GBDT) | XGBoost outperformed the other classifiers in terms of accuracy, recall, and F1-score. In addition, the performance of XGBoost was more stable than other classifiers |
| 6 | 256 primary breast carcinomas  159 had no recurrence, whereas, 97 patients presented recurrence^47^ | A Clinical Decision Support System for Predicting Invasive Breast Cancer Recurrence: Preliminary Results | Naive Bayes, RF, and SVM | The best classification performances were obtained by RF classifier |
| 7 | 158 cases who received NACT. (115 had no recurrence and 43 with recurrence)^19^ | Early prediction of breast cancer recurrence for patients treated with neoadjuvant chemotherapy: a transfer learning approach on DCE-MRIs | CNN, AlexNET as a feature extractor combined with SVM with linear kernel as classifier. | The best results were achieved when the optimal CNN features were augmented by four clinical variables (age, ER, PR, HER2+) |
| 8 | 610 primary breast carcinomas^18^ | Machine learning techniques in breast cancer prognosis prediction: A primary evaluation | ANN and SVM | Both ANN and SVM were accurate and specific to assess an individualized risk of recurrence or death from the disease |
| 9 | 922 primary breast carcinomas^14^ | Predicting disease recurrence in breast cancer patients using machine learning models with clinical and radiomic characteristics: a retrospective study | Adaptive Boosting (AdaBoost), Random Under-sampling Boosting (RUSBoost), XGBoost, SVM | The XGBoost algorithm is widely recognized as the most effective algorithm in terms of performance |
| 10 | 94 second primary malignant breast cancer (SBC)  106 recurrent breast cancer^32^ | Predicting second breast cancer among women with primary breast cancer using machine learning algorithms, a population-based observational study | Feed-forward ANN, LR, XGBoost | The ANN model had the highest area under the ROC curve. The most important features for SBC prediction were age at incidence, year of birth, stage, and extent of the pathological primary tumor |

ANN, artificial neural network; DT, decision tree; LR, logistic regression; MLP, multi-layer perceptron; SVM, support vector machine; RF, random forrest; XGBoost, Extreme Gradient Boosting
